# Synopsis of *Acalypha* (Euphorbiaceae) of continental Ecuador

**DOI:** 10.3897/phytokeys.17.3190

**Published:** 2012-09-11

**Authors:** José María Cardiel Sanz, Pablo Muñoz Rodríguez

**Affiliations:** 1 Departamento de Biología, Facultad de Ciencias, Universidad Autónoma de Madrid. Ciudad Universitaria de Cantoblanco, Postal Code 28049, Madrid, Spain

**Keywords:** *Acalypha*, Ecuador, Euphorbiaceae, lectotypification, species identification

## Abstract

A critical review of the Ecuadorian species of *Acalypha* L. (Euphorbiaceae) is presented; 20 of the 38 previously recognized species are accepted, 9 are considered synonyms and 9 are based on misidentifications. Comprehensive nomenclatural information is supplied and 13 lectotypes are designated. An identification key is also provided.

## Abstract (es)

Se presenta una revisión crítica de las especies ecuatorianas de *Acalypha* L. (Euphorbiaceae); se aceptan 20 de las 38 especies previamente reconocidas, 9 se consideran sinónimos y otras 9 están basadas en identificaciones erróneas. Se aporta una exhaustiva información nomenclatural y se designan 13 lectótipos. Se incluye también una clave de identificación.

## Introduction

*Acalypha* L. is the third largest genus in the Euphorbiaceae sensu stricto (after *Euphorbia* L. and *Croton* L.). It comprises c. 500 species found mostly in the tropics worldwide, although some reach temperate regions. The Americas are home to two thirds of the species, from southeastern Canada and United States to Uruguay and northern Argentina. They thrive in a wide variety of habitats, from tropical rainforests to subdesertic areas, and from sea level up to 4000 meters of altitude. *Acalypha* belongs to subfamily Acalyphoideae, the most diverse and complex in the Euphorbiaceae (Hayden and Hayden 2000). This subfamily appears to be paraphyletic, but the central group of taxa, Acalyphoideae Beilschm. sensu stricto, is clearly monophyletic. Molecular analysis support the monophyly of *Acalypha* (Tokuoka 2007, Wurdack and Davis 2009).

Despite its great diversity, *Acalypha* is one of the lesser known genera of the Euphorbiaceae. The last treatment of the whole genus was made by Pax and Hoffman (1924), on wich 18 species were recorded in Ecuador (Table 1). The state of the knowledge of the genus in South America includes updated national floristic treatments (Cardiel 1995, 1999, Levin 2001), and checklists (Bacigalupo and Mulgura 1999, Berry et al. 2007, Brako 1993, Cardiel 2007, 2010, Levin 2008). Regarding Ecuador, the only complete work on *Acalypha* is the treatment of Euphorbiaceae for the Catalogue of the Vascular Plants of Ecuador (Webster 1999), which recognized 34 species for the continental land of Ecuador (and four other from Galapagos Island). This work was updated by Ulloa Ulloa and Neill (2005), who added another four species of *Acalypha *, but none in the second update (Neill and Ulloa Ulloa 2011). There are also several regional or thematic floras mentioning *Acalypha* species (Bonifaz and Cornejo 2004, Cerón et al. 2007, De la Torre et al. 2008, León-Yánez et al. 2011, Madsen et al. 2001, Valencia et al. 2000). In addition Cardiel (2000) described two new species and proposed several new synonyms for Ecuadorean *Acalypha*.

This work presents a reviewed critical synopsis of the species of *Acalypha* for continental Ecuador and provides a key to help identification.

**Table 1.** Species of *Acalypha* cited for Ecuador by Pax and Hoffmann (1924), Webster (1999), and in this work; the vouchers refers to cited by Webster (1999).

Pax and Hoffmann (1924)	Webster (1999)	In this work
	*Acalypha alopecuroides* Jacq.	accepted
	*Acalypha amentacea* Roxb.subsp.* wilkesiana* (Fosb.) Müll. Arg.	= *Acalypha wilkesiana* Müll. Arg. (fide Sagun et al. 2010)
*Acalypha andina* Müll. Arg.	accepted	= *Acalypha padifolia* Kunth (fide Cardiel 2000)
	*Acalypha argomuelleri* Briq.	The voucher cited (P. Jørgensen 1551) corresponds to *Acalypha padifolia* Kunth. *Acalypha argomuelleri* has not been found in Ecuador.
	*Acalypha aronioides* Pax & K. Hoffm.	The voucher cited (P. Jørgensen 1449) corresponds to *Acalypha padifolia* Kunth. *Acalypha aronioides* has not been found in Ecuador
*Acalypha arvensis* Poepp.	accepted	accepted
	*Acalypha benensis* Britton	The voucher cited (Berg & Akkermans 1016) corresponds to *Acalypha stachyura* Pax. *Acalypha benensis* is a synonym of *Acalypha stricta* Poepp., not found in Ecuador (fide Cardiel 2007)
	*Acalypha brachyclada* Müll. Arg.	Doubtful taxon not found in Ecuador (fide Cardiel 2007)
	*Acalypha cuneata* Poepp.	accepted
	*Acalypha cuspidata* Poepp.	accepted
*Acalypha dictyoneura* Müll. Arg.	accepted	accepted
*Acalypha diversifolia* Jacq.	accepted	accepted
*Acalypha ecuadorica* Pax & K. Hoffm.	accepted	= *Acalypha schiedeana* Schltdl. (fide Cardiel 2000)
*Acalypha eggersii* Pax & K. Hoffm.	accepted	= *Acalypha cuneata* Poepp. (fide Cardiel 1995)
*Acalypha heterodonta* Müll. Arg.	= *Acalypha macrostachya* Jacq.	= *Acalypha macrostachya* Jacq. (fide Cardiel 1995)
	*Acalypha hispida* Burm. f.	accepted
*Acalypha infesta* Poepp.	not cited	accepted
	*Acalypha macbridei* I.M. Johnst.	= *Acalypha salicifolia* Müll. Arg. (fide Cardiel 2000)
	*Acalypha macrodonta* Müll. Arg.	= *Acalypha padifolia* Kunth (fide Cardiel 2007)
*Acalypha macrostachya* Jacq.	accepted	accepted
	*Acalypha mapirensis* Pax	The voucher cited (C. Ceron & C. Iguago 5461) correspond to *Acalypha stachyura* Pax. *Acalypha mapirensis* is a synonym of *Acalypha stricta* Poepp., not found in Ecuador (fide Cardiel 2007)
	*Acalypha ostryifolia* Riddell	The voucher cited (Dodson & al. 7109) corresponds to *Acalypha subcastrata* Aresch. *Acalypha ostryfolia* has not been found in Ecuador
	*Acalypha padifolia* Kunth	accepted
*Acalypha platyphylla* Müll. Arg.	accepted	accepted
*Acalypha ruiziana* Müll. Arg.	accepted	The voucher cited (Sodiro 151) corresponds to *Acalypha dictyoneura* Pax. *Acalypha ruiziana* is a synonym of *Acalypha padifolia* Kunth (fide Cardiel 2007)
*Acalypha salicifolia* Müll. Arg.	accepted	accepted
	*Acalypha scandens* Benth.	accepted
		*Acalypha schiedeana* Schltdl. (cited first time in Ecuador by Cardiel (2000)
	*Acalypha schimpffii* Diels	= *Acalypha padifolia* Kunth (fide Cardiel 2000)
*Acalypha setosa* A. Rich.	accepted	The voucher cited (Madsen 63051) corresponds to *Acalypha subcastrata* Aresch. *Acalypha setosa* has not been found in Ecuador
	*Acalypha stachyura* Pax.	accepted
		*Acalypha stellata* Cardiel (fide Cardiel 2000)
*Acalypha stellipila* Pax & K. Hoffm.	= *Acalypha dictyoneura* Müll. Arg.	= *Acalypha dictyoneura* Müll. Arg.
	*Acalypha stenoloba* Müll. Arg.	The voucher cited (Van der Werff & W. Palacios 10355) corresponds to *Acalypha stachyura* Pax. *Acalypha stenoloba* has not been found in Ecuador
	*Acalypha subandina* Ule	= *Acalypha platyphylla* Müll. Arg. (fide Cardiel 1995)
*Acalypha subcastrata* Aresch.	accepted	accepted
*Acalypha tenuipes* Pax & K. Hoffm.	accepted	= *Acalypha cuspidata* Jacq. (fide Cardiel 2000)
*Acalypha tunguraguae* Pax & K. Hoffm.	accepted	= *Acalypha cuspidata* Jacq. (fide Cardiel 2000)
*Acalypha villosa* Jacq	accepted	accepted
		*Acalypha websteri* Cardiel (fide Cardiel 2000)

## Materials and methods

We studied 987 Ecuadorian collections of *Acalypha* from the following herbaria: A, AAU, B, BM, COL, DAV, F, G, GB, GH, HAL, HBG, JE, K, L, M, MA, MO, NY, P, PR, QCA, QCNE, S, SEL, U, UPS, US, W, WRSL and Z (acronyms according to Thiers 2011). We reviewed all the collections cited by Webster (1999) and Ulloa Ulloa and Neill (2005), solving the doubts raised by some names. We also found a large number of type specimens, clarifying the identity of many names. Typifications were made after a carefully review of the original literature on the taxa, and examination of the nomenclatural types. Where no holotype was indicated, or it has been lost or destroyed, a lectotype is designated according International Code of Botanical Nomenclature rules and recommendations (McNeill et al. 2006).

The structure of the checklist follows, in general terms, those of Brako and Zarucchi (1993) and Webster (1999). The accepted species are cited in alphabetical order, including original publications, homotypic synonyms and nomenclatural synonyms based on Ecuadorian collections. For each name, the information concerning the type collections is included, with studied specimens indicated with an exclamation mark (!). Then, we summarize information about habit, habitat and altitudinal range in 250 meters intervals; this information was obtained exclusively from the studied specimens. We follow the geographic regions proposed by Jørgensen and León-Yánez (1999). They defined three regions for continental Ecuador: Coastal, Andean and Amazonian. The Coastal region is defined as below 1000 meters elevation, from west of the Andes to the coast, while the Amazonian region is defined in the same altitudinal range to the east of the Andes. The Andean region is defined for lands above 1000 meters elevation. Ecuadorian provinces where the species are recorded are cited in accordance with the studied collections, following the Ecuadorian provinces after the 2007 reorganization (i.e., including the new provinces of Santa Elena and Santo Domingo de los Tsáchilas, as well as Orellana, created in 1998). We indicate the total number of collections reviewed per taxa and one representative specimen (voucher), indicating the herbaria acronym where it is deposited. Finally we indicate post-Webster (1999) bibliographic sources which offer updated information about the species. In the “notes” section we include, when needed, any other relevant information, including justifications for nomenclatural decisions.

## Data resources

All the information gathered as part of this work is available online in the regularly updated “Acalypha Taxonomic Information System” Website (Cardiel J.M., P. Muñoz, E. Dorda & M. Pardo de Santallana**.** Acalypha Taxonomic Information System. http://www.acalypha.es ). In addition, the information of the studied specimens has been also uploaded to the Global Biodiversity Information Facility (GBIF) (http://data.gbif.org/datasets/resource/12046/ ).

## Results

Our work records 20 accepted species of *Acalypha* for continental Ecuador. Two of them are endemic: *Acalypha stellata* and *Acalypha websterii*, and two others are allochtonous: *Acalypha hispida* and *Acalypha wilkesiana*. An identification key is provided. Of the 34 species recognized by Webster (1999) for this territory, nine are considered as synonyms and nine are based on misidentifications (Table 1). The four species added by Ulloa Ulloa and Neill (2005) are accepted. We identify 17 synonyms based on Ecuadorian collections, including the new one *Acalypha pilocardia* Gilli. We indicate the type specimens of almost all the treated names, and 13 lectotypes are designated.

### Key to the species of *Acalypha* of continental Ecuador

#### Key to the subgenera

**Table:** 

1	Female flowers pedicellate; calyx with 4 or 5 sepals, the subtending bracts inconspicuous, not becoming foliaceous in fruit	Subgen. *Linostachys*
2	Female flowers sessile; calyx with 3 sepals, the subtending bracts becoming foliaceous and accrescent in fruit (except in *Acalypha hispida*)	Subgen. *Acalypha*


#### Key to species

Subgenus *Linostachys*

**Table:** 

1a	Leaf blade palmately nerved, brightly colored minute resinous droplets present, mainly on lower surface	18. *Acalypha villosa*
1b	Leaf blade pinnately nerved, brightly colored minute resinous droplets absent	2
2a	Female inflorescences paniculate. Leaf blade with 10–17 veins per side; stipules generally more than 5 mm long. Petioles more than 1 cm long	11. *Acalypha platyphylla*
2b	Female inflorescences racemose. Leaf blade with 9–13 veins per side; stipules inconspicuous, ca. 1 mm long. Petioles less than 1 cm long	12. *Acalypha salicifolia*

Subgenus *Acalypha*

**Table:** 

1a	Herb or suffrutex	2
1b	Trees or shrubs	6
2a	Female inflorescences ellipsoid or cylindrical, densely flowered, with the axis completely covered by the flowers	3
2b	Female inflorescences loosely flowered, with the axis conspicuously visible	5
3a	Female bract with long awned lobes	4
3b	Female bract with triangular awnless lobes	5. *Acalypha infesta*
4a	Young branches and leaves without glandular hairs; leaf blade acute; styles branched	2. *Acalypha arvensis*
4b	Young branches and leaves with glandular hairs; leaf blade acuminate; styles unbranched	1. *Acalypha alopecuroidea*
5a	Female inflorescences terminal, bracts with filiform lobes cut more than ½ length to the base	17. *Acalypha subcastrata*
5b	Female inflorescences axillary, bracts with triangular lobes cut ca. ¼ length to the base	4. *Acalypha cuspidata*
6a	Leaves with indumentum of stellate hairs	7
6b	Leaves without indumentum of stellate hairs	8
7a	Female inflorescences terminal, subtending bracts and styles with stellate hairs	16. *Acalypha stellata*
7b	Female inflorescences generally axillary, rarely terminal, subtending bracts and styles without stellate hairs	5. *Acalypha dictyoneura*
8a	Female or bisexual inflorescences terminal	9
8b	Female or bisexual inflorescences axillary	12
9a	Female bracts without glandular hairs	18. *Acalypha stachyura*
9b	Female bracts with glandular hairs	10
10a	Female bracts subtriangular at maturity, with the central tooth prominent, lanceolate, acuminate	19. *Acalypha websteri*
10b	Female bracts suborbicular at maturity, with the central tooth not or slightly prominent	11
11a	Leaf blade generally broadly ovate-lanceolate; accrescent bracts with glandular hairs ca. 0.3–0.5 mm long; styles 4–5 mm long.	14. *Acalypha schiedeana*
11b	Leaf blade generally narrowly ovate-lanceolate; accrescent bracts with glandular hairs ca. 1 mm long; styles 7–8 mm long	10. *Acalypha padifolia*
12a	Plants with both unisexual and bisexual inflorescences	13
12b	Plants with all the inflorescences unisexual	14
13a	Leaf blade generally triangular-lanceolate, palmately nerved	4. *Acalypha cuspidata*
13b	Leaf blade elliptic-lanceolate or oblong-lanceolate, pinnately nerved	6. *Acalypha diversifolia*
14a	Leaf blade pinnately nerved	15
14b	Leaf blade palmately nerved	16
15a	Leaf blade generally obovate, the base subcuneate; female inflorescences 7–15 cm long	3. *Acalypha cuneata*
15b	Leaf blade ovate to oblong-lanceolate, the base rounded to subcordate; female inflorescences 25–40 cm long	12. *Acalypha scandens*
16a	Female inflorescences extremely densely flowered, with the axis hidden; bracts non-accrescent	9. *Acalypha hispida*
16b	Female inflorescences more or less densely flowered, with the axis visible; bracts conspicuously accrescent	17
17a	Leaf blade generally variegated; female inflorescences up to 10 cm long	20. *Acalypha wilkesiana*
17b	Leaf blade not variegate; female inflorescences more than 15 cm long	9. *Acalypha macrostachya*

### Catalogue to the species of *Acalypha* of continental Ecuador

01. ***Acalypha alopecuroidea*** Jacq., Collectanea 3: 196. 1789[1791].

**Type.** Crescit in Venezuela, tab. 620 in Jacq., Ic. Pl. Rar. 3 (1792), lectotype designated by Cardiel, Anales Jard. Bot. Madrid 54: 233 (1995[1996]).

≡ *Ricinocarpus alopecuroides* (Jacq.) Kuntze, Revis. Gen. Pl. 2: 617. 1891.

Herb. Coastal, 0–250 m. ‒ Provincial distribution: Guayas (2 collections examined). ‒ Voucher: F.M.Valverde 334 (COL, US).

**References.** Levin (2001), Madsen et al. (2001), Cardiel (2007).

**Note.** We found only two Ecuadorian collection of this species, which is widely distributed in Central America, Venezuela and Colombia.

02. ***Acalypha arvensis*** Poepp. in Poepp. Endl., Nov. Gen. Sp. Pl. 3: 21. 1841.

**Type.** [PERU] Crescit in cultis et ruderalis provinciae Maynas ad Yurimaguas, toto anno florens, E.Poeppig 2215[2115] (**lectotype:** W!, **designated here**; isolectotypes G[2 sheets]!, F[fragment ex W]!, W!). Other type collection: [BRAZIL] ad Serpa in provincia Paranaensi, E.Poeppig s.n. (W!).

≡ *Ricinocarpus arvensis* (Poepp.) Kuntze, Revis. Gen. Pl. 2: 617. 1891.

Annual herb or small suffrutex. Coastal, 0–1000 m. Roadsides and disturbed vegetation. ‒ Provincial distribution: Cotopaxi, Guayas, Los Ríos, Manabí, Napo, Pichincha, Sucumbíos (14 collections examined). ‒ Voucher: O.Haught 2994 (GH, US).

**References.** Levin (2001), Cardiel (2007).

**Note.** *Acalypha arvensis* was described based on two collections: E.Poeppig 2215 and E.Poeppig s.n., from Peru and Brazil respectively. We select one of the two specimens of the collection E.Poeppig 2215 found in the W herbarium, as lectotype. Some duplicates of this collection from G and W herbaria also show an original label with the number 2115, in addition of 2215.

03. ***Acalypha cuneata*** Poepp., Nov. Gen. Sp. Pl. 3: 22. 1841.

**Type.** [PERU] Crescit in fruticetis maynensibus ad Yurimaguas. Martio lecta, E.Poeppig 2230 (**lectotype:** W[113778]!, **designated here**; isolectotypes, B[destroyed, photo 5288], F!, W!). Other type collections: E.Poeppig 2317 (B[destroyed, photo 5288], F!, G!, P[2 sheets]!, W!), 2330 (F!, G[4 sheets]!, P[2 sheets]!, W!), 2807 (W!).

≡ *Ricinocarpus cuneatus* (Poepp.) Kuntze, Revis. Gen. Pl. 2: 617. 1891; *Acalypha obovata* Benth. var. *cuneata* (Poepp.) J.F. Macbr., Candolea 6: 26. 1940.

= *Acalypha obovata* Benth., Bot. Voy. Sulphur. 163, tab.53. 1846; *Acalypha cuneata* Poepp. var. *obovata* (Benth.) Müll. Arg., Linnaea 34: 14. 1865. **Type:** [ECUADOR, Esmeraldas] Atacames, tab. 53 in Benth., loc. cit. 1846.

= *Acalypha eggersii* Pax, Bot. Jarhb. Syst. 26: 205. 1899. **Type:** Ecuador: prov. Manabí, prope Hacienda El Recreo, H.Eggers 15007 (**lectotype:** S!, **designated here**; isolectotypes: B[destroyed, photo 5291], C!, F[2 sheets]!, MA!, MO!, NY!, US!).

Shrub or small tree. Mainly Amazonian and Coastal, excepcionally Andean, (0-) 250–1000(-1500) m. Generally associated with primary rainforests. ‒ Provincial distribution: Esmeraldas, Guayas, Los Ríos, Manabí, Morona-Santiago, Napo, Ore-llana, Pastaza, Santa Elena, Santo Domingo de los Tsáchilas , Sucumbíos, Tungurahua, Zamora-Chinchipe (222 collections examined). ‒ Voucher: E. Gudiño 61 (GB, DAV, MO, QCNE, U).

**References.** Bonifaz and Cornejo (2004), Cardiel (2007), De la Torre et al. (2008), Santiana and Cerón (2000).

**Notes.** Poeppig described *Acalypha cuneata* based on four Peruvian collections: E.Poeppig 2230, 2317, 2330 and 2807. We selected the best preserved specimen, in the W herbarium, as lectotype. The synonym *Acalypha eggersii* Pax was described from a single collection (F.A.Eggers 15007), which was distributed to several herbaria; due to the destruction of Berlin specimen, we designate as lectotype the specimen from the S herbarium.

04. ***Acalypha cuspidata*** Jacq., Pl. Hort. Schoenbr. 2: 63, tab. 243. 1797.

**Type.** [VENEZUELA] Crescit ad Caracas, tab. 243 in Jacq., loc. cit. 1797, lectotype designated by Cardiel, Anales Jard. Bot. Madrid 54: 233 (1995[1996]).

= *Acalypha vestita* Benth., Bot. Voy. Sulpuhur. 164. 1844. **Type:** [ECUADOR] Guayaquil, Sinclair s.n. (K[in hb. Hook.]).

= *Acalypha tenuipes* Pax & K. Hoffm. in Engl., Pflanzenr. 147, 16 (heft. 85): 122. 1924. **Type:** Ecuador trockene Gebüsche bei Agua Amarga, Elrecreo, H.Eggers 15833 (**lectotype:** US!, **designated here**; isolectotypes: B[destroyed, photo 5325], F!, K! [2 sheets], L!, M!).

Shrub or suffrutex. Coastal, 0–250 m. Associated with dry deciduous forest, savanna and thickets. ‒ Provincial distribution: El Oro, Guayas, Manabí, Santa Elena (8 collections examined). ‒ Voucher: J.E. Madsen 63938 (AAU, MA, QCA, QCNE).

**References.** Cardiel (2000, 2007), Madsen et al. (2001), De la Torre et al. (2008).

**Notes.** *Acalypha cuspidata* Jacq. is often confused with *Acalypha plicata* Müll. Arg., which has a conspicuous glandular indumentum on the young branches, leaves and inflorescences. *Acalypha plicata* is frequent in the Andean zones of Colombia and Peru, but has not been found in Ecuador where it is also likely to be present. The synonym *Acalypha tenuipes* Pax & K. Hoffm. was described from a single collection (H.Eggers 15833), which was distributed to several herbaria; due to the destruction of Berlin specimen, we select as lectotype the best preserved and most complete specimen found in the US herbarium. See comments about this synonym in Cardiel (2000).

05. ***Acalypha dictyoneura*** Müll. Arg., Linnaea 34: 12. 1865.

**Type.** [PERU] In Peruvia prope Chachapoyas, A.Matthews s.n. (holotype: G!; isotype: K!).

= *Acalypha pilocardia* Gilli, Feddes Repert. Spec. Nov. Regni Veg. 92: 678. 1981. **Syn. nov.** **Type:** [ECUADOR] Schlucht des Angamarca-Flusses bei El Corazón, 1300 m, 1.7.75, 301, fl., A.Gilli 301 (holotype: W!).

= *Acalypha stellipila* Pax & K. Hoffm. in Engl., Pflanzenr. 147, 16 (heft. 85): 49. 1924. **Type:** Ecuador, Gualea, Sodiro 151/26^a^ (**lectotype:** F[644679]!, **designated here**; isolectotype B[destroyed, photo 71872]). Other type collections: Ecuador, Puente de Chimbo, Sodiro 151/26^b^ (B[destroyed, photo 5321]).

Shrub or small tree. Andean, (1300)1750–2500 m. Montane rainforests. ‒ Provincial distribution: Bolívar, Chimborazo, Imbabura, Napo, Orellana, Pichincha, Santo Domingo de los Tsáchilas, Tungurahua (52 collections examined). ‒ Voucher: M.Balslev & E.Madsen 10362 (AAU, COL, C, F, MO, NY, QCA, SEL, US).

**References.** Cardiel (2000, 2007), Santiana and Cerón (2000).

**Note.** The synonym *Acalypha stellipila* Pax & K. Hoffm. was described from two collections (Sodiro 151/26^a^ and 151/26^b^), both destroyed in B herbarium. We designate as lectotype a fragment of the first one, preserved in F herbarium.

06. ***Acalypha diversifolia*** Jacq., Pl. Hort. Schoenbr. 2: 63, tab. 244. 1797.

**Type.** [VENEZUELA] ex Caracas, tab. 244 in Jacq., Pl. Hort. Schoenbr. 2 (1797), lectotype designated by Cardiel, Anales Jard. Bot. Madrid 54: 233 (1995[1996]).

Shrub or small tree. Amazonian, Andean and Coastal, 0–2000 m. Lowland rainforests, deciduous and semi-deciduous forests (often along river banks) and disturbed areas. ‒ Provincial distribution: Bolívar, Carchi, Chimborazo, Cotopaxi, El Oro, Esmeraldas, Guayas, Imbabura, Loja, Los Ríos, Manabí, Morona-Santiago, Napo, Orellana, Pastaza, Pichincha, Santo Domingo de los Tsáchilas, Sucumbíos, Tungurahua, Zamora-Chinchipe (310 collections examined). ‒ Voucher: V.Zak & J.Jaramillo 2822 (DAV, F, GB, GH, K, MO, NY, QCNE, SEL).

**References.** Levin (2001), Cardiel (2007), De la Torre et al. (2008).

07. ***Acalypha hispida*** Burm. f., Fl. Ind. 302, tab. 61, f. 1. 1768.

**Type.** Habitat in India, tab. 61 in Burm., loc. cit. 302. 1768.

Shrub. Coastal, 0–250 m. Cultivated and naturalized. ‒ Provincial distribution: Guayas, Santo Domingo de los Tsáchilas (4 collections examined). ‒ Voucher: L.P.Kvist & E.Asanza 40725 (AAU, QCA, QCNE).

**References.** Levin (2001), Cardiel (2007), De la Torre et al. (2008).

**Note.** Native to Malaysia or Melanesia, this species is grown in gardens throughout the tropics, and sometimes appears naturalized.

08. ***Acalypha infesta*** Poepp. in Poepp. & Endl., Nov. Gen. Sp. Pl. 3: 22. 1845.

**Type.** [PERU] Crescit in cultis ad Cuchero, E.Poeppig 1701 (**lectotype:** W[103476]!, **designated here**; isolectotypes: F!, P!, US!, W!).

Annual herb or suffrutex. Andean, 1000–2500 m. Disturbed areas. ‒ Provincial distribution: Bolívar, Cañar, Chimborazo, Pichincha (6 collections examined). ‒ Voucher: F.R.Fosberg & M.A.Giler 22627 (NY, US).

**References.** Cardiel (2001, 2007), Ulloa Ulloa and Neill (2005).

**Notes.** This species is often confused with *Acalypha poiretii* Spreng., present in South America (mostly in Argentina and Bolivia), and not found in Ecuador. *Acalypha poiretii* has bisexual inflorescences, the calyx of female flowers with four sepals and female bracts with smaller teeth. *Acalypha infesta* Poepp. was described from a single collection (E.Poeppig 1701), which was distributed to several herbaria. We select as lectotype one of the two sheets conserved in the W herbarium.

09. ***Acalypha macrostachya*** Jacq., Pl. Hort. Schoenbr. 2: 63, t. 245. 1797.

**Type.** [VENEZUELA] crescit ad Caracas, tab. 245 in Jacq., Pl. Hort. Schoenbr.: 2 (1797), lectotype designated by Cardiel, Anales Jard. Bot. Madrid 54: 233 (1995[1996]).

= *Acalypha heterodonta* Müll. Arg. var *hirsuta* Müll. Arg., Linnaea 34: 12. 1865. **Type:** [Ecuador] Ad pedem montis Chimborazo, R.Spruce 6147 (holotype: K!; isotypes: BM!, W!).

Shrub or small tree. Amazonian, (250-)500–2000 m. In lowland and lower montane rainforests, frequent in disturbed areas. ‒ Provincial distribution: Carchi, Chimborazo, El Oro, Esmeraldas, Morona-Santiago, Napo, Orellana, Pastaza, Pichincha, Santo Domingo de los Tsáchilas, Sucumbíos, Tungurahua, Zamora-Chinchipe (103 collections examined). ‒ Voucher: H.Balslev & E.Madsen 10462 (AAU, C, COL, F, GB, MO, NY, QCA, SEL, US).

**References.** Levin (2001), Cardiel (2007), Cerón et al. (2007), De la Torre et al. (2008).

10. ***Acalypha padifolia*** Kunth in Humb. & Bonpl., Nov. Gen. Sp. (quarto ed.) 2: 97. 1817.

**Type.** [COLOMBIA] Crescit locis sylvaticis subfrigidis inter Almaguer et Pasto, prope villam Meneses, alt. 1322 hex, A.Humboldt & A.Bonpland 2136 (holotype: P-Bonpl.!; isotype: P!).

= *Acalypha andina* Müll. Arg., Flora 55: 26. 1872. **Type:** [ECUADOR] In andibus orientalibus ecuadorensibus inter Bannas et Rio verde altitudine circ. 5000 ped., M.Wagner s.n. (holotype: M!).

= *Acalypha tunguraguae* Pax & K. Hoffm. in Engl., Pflanzenr. 147, 16 (heft. 85): 66. 1924. **Type:** Ecuador, am vulkan Tunguragua, 1800–2000 m., F.Lehmann 6641 (**lectotype:** K[600517]!, **designated here**; isolectotype: B[destroyed, photo 5327]).

= *Acalypha schimpffii* Diels, Biblioth. Bot. 29: 103. 1937. **Type:** Mittel-Ecuador: West-Kordillere: Prov. Chimborazo: Tal des R. Chanchan bei Huigra an buschigen Abnhägen, 1260 m. 16 November, H.J.F.Schimpff 429 (**lectotype:** MO!, **designated here**; isolectotypes: A[3 sheets]!, M!, NY, US!, Z[3 sheets]!). Other type collection: Ecuador, loc. cit. 21 September 1993, Diels 1128 ([B destroyed]).

Shrub or small tree. Andean, 2000–3250(3800) m. Upper and lower montane rainforests, mainly disturbed. ‒ Provincial distribution: Azuay, Bolívar, Cañar, Carchi, Chimborazo, Imbabura, Loja, Pastaza, Pichincha, Tungurahua (83 collections examined). ‒ Voucher: C.E.Cerón & G.Benavides 2551 (F, MO, QCA, QCNE).

**References.** Cardiel (2000, 2007), De la Torre et al. (2008), Santiana and Cerón (2000).

**Notes.** The synonym *Acalypha tunguaraguae* Pax & K. Hoffm. was described from a single collection (H. Eggers 15833); due to destruction of the Berlin specimen, we select as lectotype the duplicate found in the K herbarium. The synonym *Acalypha schimpffii *Diels was described based on two collections: Diels 1128, destroyed in B herbarium, and Schimpff 429, poorly preserved, which was distributed to several herbaria; we select as lectotype the specimen preserved in the MO herbarium.

11. ***Acalypha platyphylla*** Müll. Arg., Linnaea 34: 6. 1865.

**Type.** In Ecuador Peruviae, L.Fraser s.n. (holotype: G-DC[324093]!).

≡ *Ricinocarpus platyphyllus* (Müll. Arg.) Kuntze, Revis. Gen. Pl. 2: 618. 1891.

Shrub or small tree. Andean, 1250–2500 m. In lower and upper montane rain- forests. ‒ Provincial distribution: Azuay, Cotopaxi, Morona-Santiago, Napo, Orellana, Pichincha, Santo Domingo de los Tsáchilas (87 collections examined). ‒ Voucher: J.Jaravillo & V.Zak 7481 (F, MO, NY, QCA, QCNE).

**References.** Cardiel (2007).

12. ***Acalypha salicifolia*** Müll. Arg., Flora 47: 438. 1864.

**Type.** [ECUADOR] in Andibus Ecuadorensibus, R.Spruce 4963 (holotype: W!; isotypes, F, K[2 sheets, fragments ex W]!).

≡ *Ricinocarpus salicifolius* (Müll. Arg.) Kuntze, Revis. Gen. Pl. 2: 618. 1891.

Shrub or small tree. Amazonian, 500–1250(-1750) m. In lower montane rainforests and lowland rainforests. ‒ Provincial distribution: Morona-Santiago, Napo, Sucumbíos, Zamora-Chinchipe (28collections examined). ‒ Voucher: H.Van der Werff & E.Gudiño 11350 (G, GB, MO, NY, QCNE).

**References.** Cardiel (2000, 2007), Cerón et al. (2007), De la Torre et al. (2008).

13. ***Acalypha scandens*** Benth., J. Bot. (Hooker) 6: 329. 1854.

**Type.** [BRAZIL] On the island of the Amazon opposite Santarem, R.Spruce 1000 (holotype: K!).

Shrub or small tree. Amazonian, 0–250 m. Lowland rainforests, often along river banks. ‒ Provincial distribution: Orellana, Pastaza, Sucumbíos (6 collections examined). ‒ Voucher: C.E.Cerón et N.Gallo 5186 (AAU, MO, QCNE).

**References.** Cardiel (2007).

14. ***Acalypha schiedeana*** Schltdl., Linnaea 7: 384. 1832.

**Type.** [MEXICO] In sylvis umbrosis Jalapae, C.J.W. Schiede 44 (**lectotype:** P[645420]! **designated here**). Other type collections: C.J.W.Schiede 72 (B[destroyed, photo 5319]!), 247 (HAL[072241]!).

= *Acalypha ecuadorica* Pax & K. Hoffm. in Engl., Pflanzenr. 147, 16 (heft 85): 68. 1924. **Type:** Ecuador, Agua Amarga bei El recreo, trockene Gebüsche, H.Eggers 15535 (**lectotype:** S[S-R-7770]!, **designated here**; isolectotypes: B[destroyed, photo 5290], BM, C!, F!, GH, K[2 sheets]!, L!, M!, MA!, NY!, PR, US!).

Shrub or small tree. Coastal, 0–500(-750) m. Generally associated with dry forests and thickets. ‒ Provincial distribution: Guayas, Loja, Los Ríos, Manabí (19 collections examined). ‒ Voucher: E.Asplund 15240 (S)

**References.** Cardiel (1999, 2000), Santiana and Cerón (2000), Ulloa Ulloa and Neill (2005).

**Notes.** *Acalypha schiedeana* Schltdl. was described from a single collection, Schiede 72. The specimen deposited in the B herbarium was destroyed. We select as lectotype the duplicate found in the K herbarium. The synonym *Acalypha ecuadorica* Pax & K. Hoffm. was described from a single collection, H.Eggers 15535, distributed to many herbaria. It represents young branches with immature inflorescences. Because of the destruction of the Berlin specimen, we select as lectotype the duplicate from S herbarium, which has the most developed flowers. See comments about this synonym in Cardiel (2000).

15. ***Acalypha stachyura*** Pax, Repert. Spec. Nov. Regni Veg. 7: 110. 1909.

**Type.** [BOLIVIA] Charopampa und San Carlos bei Mapiri, 750 m –August und November 1909. O.Buchtien 1315 (**lectotype:** M!, **designated here**; isolectotype US!). Other type collections: O.Buchtien 1307 (WRSL, US!), 1314 (US!).

Shrub or small tree. Amazonian region, 0–1000(-1250) m. In lowland rainforests. ‒ Provincial distribution: Morona-Santiago, Napo, Orellana, Pastaza, Sucumbíos, Zamora-Chinchipe (136 collections examined). ‒ Voucher: J.Zaruma & al. 126 (F, GB, MO, NY, QCNE).

**References.** Cardiel (2007), Cerón et al. (2007), De la Torre et al. (2008).

**Note.** *Acalypha stachyura* Pax was described based on three collections of O.Buchtien: 1307, 1314 and 1315, all from the same locality. We select as lectotype the best preserved collection, from the M herbarium.

16. ***Acalypha stellata*** Cardiel, Novon 10(4): 362. 2000.

**Type.** ECUADOR, Bolívar: Limón, estribaciones de la Cordillera Occidental, 880–1100 m, 14 Oct. 1943, M.Acosta Solís 6639 (holotype: F!; isotype: F!). Shrub or small tree. Coastal, 500–1000 m. Lower western slopes of the Andes. ‒ Provincial distribution: Bolívar, Chimborazo, El Oro (5 collections examined).

**References.** Cardiel (2000), Ulloa Ulloa and Neill (2005).

**Note.** Ecuadorian endemic.

17. ***Acalypha subcastrata*** Aresch., Pl. Itin. Eugeniae: 137. 1910.

**Type.** [ECUADOR] On Puna i Guayaquil viken, N.J.Andersson 160 (**lectotype:** S[S-R-7773]!, **designated here**; isolectotype: S[08-1622]!).

Annual herb. Coastal, 0–500(-1200) m. Generally associated with dry forest and open sites. ‒ Provincial distribution: Guayas, Loja, Los Ríos, Manabí, Santa Elena (24 collections examined). ‒ Voucher: C.M.Dodson & P.M.Dodson 13680 (MO, QCNE, SEL).

**References.** Cardiel (2007).

**Notes.** *Acalypha subcastrata* Aresch. is often confused with *Acalypha setosa* A. Rich, which has the ovary hispid and female bracts without glandular hairs (vs. ovary glabrous and bracts with glandular hairs). *Acalypha setosa* occurs in Venezuela and Colombia, and has not been found in Ecuador. *Acalypha subcastrata* was described based on a single collection, N.J.Andersson 160, of which we found two sheets in S herbarium. We select one of them as the lectotype.

18. ***Acalypha villosa*** Jacq., Enum. Syst. Pl. 32.1760.

**Type.** [COLOMBIA] Habitat Carthagenae in silvis & sepibus, tab.183, fig. 16 in Jacq., Select. Stirp. Amer. Hist. (1763). Lectotype designated by R.A. Howard (1776). Epitype: tab. 47 in Jacq., Hort. Bot. Vindov. (1776), designated by Cardiel, Anales J. Bot. Madrid (1995[1996]).

= *Acalypha villosa* Jacq. var. *latiuscula* Pax. & K. Hoffm. in Engl., Pflanzenr. 147, 16 (heft. 85): 17. 1924. **Type:** Ecuador, Manabí, bei Hacienda El Recreo, H.Eggers 15616 (**lectotype:** K[600533]!, **designated here**; isolectotype: L!). Other type collections: Ecuador, Manabí, bei Hacienda El Recreo, H.Eggers 15047(F!, K!, L!, M!, US!); Brasilien, Alto Amazonas, Rio Acre, Seringal S. Francisco, E.Ule 9537 (K!); [BRAZIL] Matto Grosso, Malme 2543; [BRAZIL] Madeira Fälle, H.Rusby 1272 (K!, NY!, US!); [BOLIVIA] Bolivien, am Zusammenfluß des Beni und Madre de Dios, H.Rusby 1271 (BM!, MA!, K!, MO!, NY!, US).

Shrub or small tree. Coastal, 0–250 m. Generally associated to secondary woods and thickets, also with savanna and deciduous forest, mainly on sandy and humid soils. ‒ Provincial distribution: El Oro, Esmeraldas, Guayas, Los Ríos, Manabí, Pichincha, Santo Domingo de los Tsáchilas (54 collections examined). ‒ Voucher: L.P.Kvist 40466 (AAU, MO, NY, QCA).

**References.** Levin (2001), Cardiel (2007), De la Torre et al. (2008).

**Note.** The synonym *Acalypha villosa var. latiuscula* Pax & K. Hoffm. was described based on six different collections from Bolivia, Brazil and Ecuador. We select as lectotype the quite well preserved Ecuadorian collection H.Eggers 15616, from the K herbarium.

19. ***Acalypha websteri*** Cardiel, Novon 10(4): 360. 2000.

**Type.** ECUADOR, Prov. Chimborazo, Huigra, ca. 1200 m, E.Asplund 15427 (holotype S!, isotype S!). Shrub. Andean, 1200 m. ‒ Provincial distribution: Chimborazo (3 collections examined). ‒ Voucher: J.E.Madsen 36847 (AAU).

**References.** Cardiel (2000), Ulloa Ulloa and Neill (2005).

**Note.** Ecuadorian endemic.

20. ***Acalypha wilkesiana*** Müll. Arg. in DC., Prodr. 15(2): 817. 1866.

**Type.** [FIJI ] In insulis Fidji (U.S. Expl. Exped. Under. Capt. Wilkes), B.C.Seeman 22 (holotype, G-DC!; isotypes, GH!, K[2]!, US[2]!).

≡ *Acalypha amentacea* Roxb. subsp. *wilkesiana* (Müll. Arg.) Fosberg, Smithsonian Contr. Bot. 45: 10. 1980.

Shrub. Cultivated and naturalized. Coastal, Andean and Amazonian, 0–1500 m. ‒ Provincial distribution: Guayas, Imbabura, Manabí, Napo, Santo Domingo de los Tsáchilas (6 collections examined). ‒ Voucher: M.Acosta-Solís 13076 (F).

**References.** Levin (2001), Cardiel (2007), Sagun et al. (2010).

**Note.** Native to the Polynesian island of Fiji , this species is widely used as an ornamental in the tropics. It has been treated as a subspecies of *Acalypha amentacea* Roxb. because of its morphological similarities (Fosberg and Sachet 1980), but a molecular study by Sagun et al. (2010) showed that it was closer to *Acalypha hispida* Burm. than to *Acalypha amentacea*, and therefore should be the species rank.
